# Is laminectomy and fusion the better choice than laminoplasty for multilevel cervical myelopathy with signal changes on magnetic resonance imaging? A comparison of two posterior surgeries

**DOI:** 10.1186/s12891-020-03435-7

**Published:** 2020-07-02

**Authors:** Xin He, Jia Nan Zhang, Tuan Jiang Liu, Ding Jun Hao

**Affiliations:** Department of Spine Surgery, Hong Hui Hospital, Xi’an Jiaotong University, No. 76 Nanguo Road, Nanshao gate, Xi’an, 710054 Shaan’xi Province China

**Keywords:** Cervical spondylotic myelopathy, Magnetic resonance imaging, Laminoplasty, Laminectomy, Fusion

## Abstract

**Background:**

This study aimed to compare the clinical outcomes and complications between laminectomy and fusion (LF) and laminoplasty (LP) for multi-level cervical spondylotic myelopathy (MCSM) with increased signal intensity (ISI) on T2-weighted images (T2WI).

**Methods:**

In this retrospective cohort study, we analyzed 52 patients with MCSM with ISI on T2WI who underwent laminoplasty (LP group). The Japanese Orthopedic Association (JOA) score, the Visual Analogue Scale (VAS) score, the physical and mental component scores (PCS and MCS) of Short-Form 36 (SF-36), and the extension and flexion ranges of motion (ROMs) were recorded. As controls, propensity score matching identified 52 patients who underwent laminectomy and fusion (LF group) from January 2014 to June 2016 using 7 independent variables (preoperation): age, sex, JOA score, SF-36 PCS, SF-36 MCS, preoperative symptom duration and high signal intensity ratio (HSIR).

**Results:**

The operative duration in the LF group was significantly higher than that in the LP group. At the last follow-up, the JOA score, VAS score, and SF-36 (PCS and MCS) scores were all significantly improved in both groups. The extension and flexion ROMs were decreased in both groups but significantly better in the LP group than in the LF group. Both groups demonstrated similar clinical improvements at the final follow-up. The complication rate was higher in the LF group.

**Conclusion:**

The present study demonstrates that LP for MCSM with ISI on T2WI achieves similar clinical improvement as LF. However, longer operative durations, higher complication rates and lower extension and flexion ROMs were found in the LF group.

## Background

Cervical spondylotic myelopathy (CSM) is an age-related degenerative disease of the cervical spine, including intervertebral disc herniation, Luschka joint hyperplasia, vertebral posterior osteophyte, and ossification of the posterior longitudinal ligament (OPLL), which cause spinal stenosis and secondary nerve root compression, resulting in spinal compression or spinal cord ischemia and secondary nerve dysfunction [1]. Multilevel cervical spondylotic myelopathy (MCSM) often has a long disease course, involves severe cervical spine degeneration, and rapidly progresses [2]. In some cases, MCSM is associated with cervical canal stenosis, severe spinal cord compression, and a long disease course, which can lead to signal changes in the spinal cord on magnetic resonance imaging (MRI). Currently, there are no specific drugs for the treatment of CSM; steroids and dehydration medicines are often used for conservative treatment but are often ineffective; thus, the main treatment for patients with severe MCSM is surgery. Posterior laminectomy and fusion (LF) and laminoplasty (LP) are both common treatments for MCSM. The advantages of LF are considered to be adequate for decompression and restoring partial physiological curvature of the cervical spine. However, compared with LF, LP also could achieve satisfactory decompression, and even preservation of the complete posterior structure and cervical motion, improve the postoperative quality of life and the early rehabilitation of patients. The advantages and disadvantages of the two surgical methods have been widely studied, that both methods can achieve satisfactory clinical outcomes, but LP had fewer complications and lower blood loss [3–5]. However, in the case of MCSM with increased signal intensity (ISI) on T2-weighted imaging (T2WI), a question is raised: does preserving cervical vertebral mobility affect the recovery of neurological function? In particular, ISI on T2WI is mainly associated with localized spinal cord edema, neuronal degeneration, spinal cord softening, and cystic necrosis after the long-term application of mechanical stress. ISI on T2WI is an irreversible pathological condition [6, 7]. Studies have suggested that the increased signal intensity is closely related to the prognosis of patients, generally indicating a poor prognosis [8, 9]. And patient with ISI on T2WI underwent laminoplasty has been reported may be associated with poor surgical outcomes [10]. The purpose of this study was to compare the clinical outcomes and complications of these two methods for MCSM with ISI on T2WI.

## Methods

### Patient selection

In this single-center, retrospective study, we analyzed 52 patients with MCSM with ISI on T2WI who underwent laminoplasty (LP group) from January 2014 to June 2016. As controls, propensity score matching identified 52 patients who underwent laminectomy and fusion (LF group) (1:1) from January 2014 to June 2016 using 7 independent variables (preoperation): age, sex, Japanese Orthopedic Association (JOA) score, Short Form 36 (SF-36) physical component score (PCS), SF-36 mental component score (MCS), preoperative symptom duration and high signal intensity ratio (HSIR).

The patients were chosen according to the following inclusion and exclusion criteria. Inclusion criteria: (1) Features conformed to the diagnostic criteria of MCSM [11, 12]; (2) MRI examination of the cervical spine showed ISI on T2WI; (3) age > 18 years; (4) surgery performed by the same surgical team; and (5) positive ‘K-line’ [13]. Exclusion criteria: (1) Cervical congenital malformations and syringomyelia; (2) cervical cancer; (3) ankylosing spondylitis or traumatic injury; (4) cervical kyphosis; (5) decreased signal intensity (DSI) on T1-weighted imaging (T1WI); (6) other severe major organ dysfunction; (7) cervical spine instability (X-ray examination of the cervical spine in flexion and extension showing horizontal displacement of two adjacent vertebrae > 3 mm and/or an angle difference > 11° between two adjacent vertebral spaces); and (8) previous history of cervical surgery.

The diagnostic criteria of MCSM based on physical and radiographic examinations were as follows: The relevant symptoms included numb hands, clumsy hands, impaired gait, bilateral arm paresthesia, Lhermitte phenomena, and weakness; the patient had least one clinical sign of myelopathy; the relevant signs of myelopathy included corticospinal distribution of motor deficits, atrophy of intrinsic hand muscles, hyperreflexia, positive Hoffman sign, positive Babinski sign, lower limb spasticity, and broad-based unstable gait; MRI examination of the cervical spine showed at least 3 levels of cervical spinal cord compression.

### Radiographic and clinical evaluations

X-ray (anteroposterior, flexion, and extension positions), MRI, and computed tomography (CT) examinations were performed before the operation, and X-ray (same positions) and CT examinations were performed at the final follow-up visit. The JOA score, the Visual Analogue Scale (VAS) score, and SF-36 PCS and MCS were recorded before the operation, 12 months after the operation, and at the final follow-up visit. The extension and flexion ranges of motion (ROMs) were recorded before the operation and at the final follow-up visit. In this method, the angle between 2 lines drawn parallel to the posterior surface of the C7 and C2 vertebral bodies was measured [14]. The aforementioned indexes were determined for both groups and compared. Moreover, the operative level, operative duration, blood loss, and surgery-related complications were also recorded and compared.

The intramedullary ISI on T2WI of each patient was recorded before the operation. The MRI data of all patients were analyzed using the image processing software ImageJ (National Institutes of Health, USA). The integrated optical density (IOD) was measured at the location of interest with an area of 0.1 cm^2^. In the same sagittal plane, the same area was used to measure the IOD at the C7/T1 level with a normal intramedullary signal, and the HSIR was calculated.

Furthermore, a CT scan was performed after each operation. Intraoperative adjustments and screws breaching the bone cortex in any direction by more than 2 mm were defined as unsatisfactory. C5 nerve root paralysis was diagnosed by deltoid weakness, brachialgia, and numbness after the operation [15]. Infection was diagnosed by postoperative fever and increases in the erythrocyte sedimentation rate, C-reactive protein level and white blood cell count after the operation or a positive bacterial wound culture. Axial symptoms (AS) were defined as pain from the nuchal to the periscapular or shoulder region after the operation [16].

### Surgical procedures

From January 2014 to June 2016, MCSM patients with ISI on T2WI underwent LP or LF. LP was performed for patients without kyphosis and/or cervical spine instability and/or needing bilateral foraminotomy. LF was performed for patients with kyphosis and/or cervical spine instability and/or needing unilateral foraminotomy. In general, the decision to pursue either LF or LP was made on a case-by-case basis, and patient wishes were taken into account if they fit the indications for both procedures. All patients provided written informed consent.

Each patient was in a prone position on a carbon fiber table. During the entire course of the surgery, neurological function was monitored by somatosensory and motor evoked potentials.

**Fig. 1:**
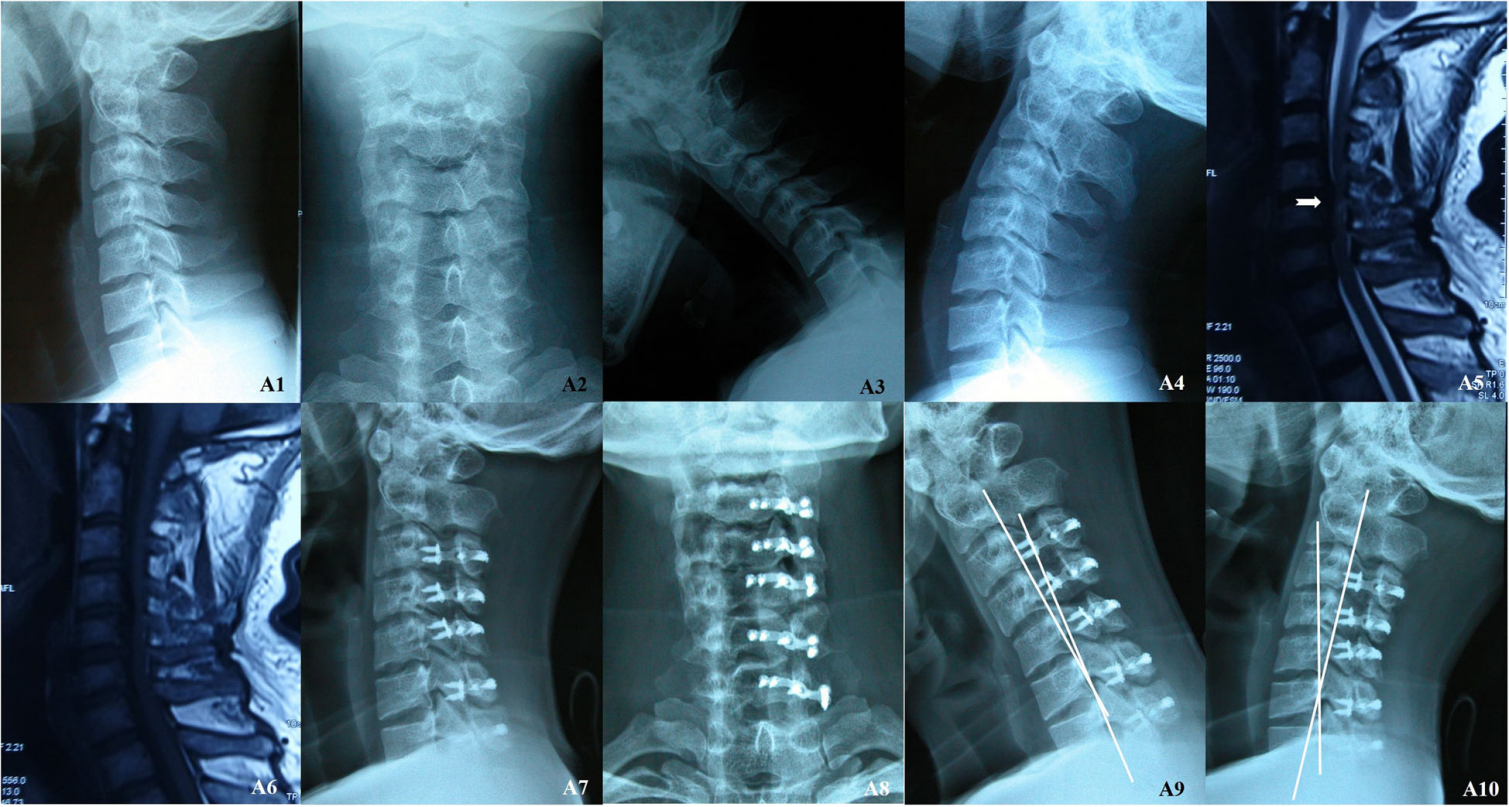
M, 42Y, multi-level cervical spondylotic myelopathy. Preoperative X-Ray images (A1 and A2); X-Ray images of extension and flexion (A3 and A4) shown no obvious cervical instability; Preoperative MRI scans (A5-A6) shown increased signal intensity on T2-weighted image (White arrow); Postoperative X-Ray images (A7 and A8); X-Ray images of extension and flexion at the 30 months after operation (A9 and A10) (White lines)

Patients in the LP group underwent LP using a standard midline posterior approach, and the decompression range was determined by the neurological symptoms and imaging examination findings. Osteotomy was performed at the level of the junction between the lamina and the lateral mass on the opening side (the right or left side was based on the laterality of each patient’s symptoms). On the opposite side, a trough was opened as a hinge. After the opening-side lamina was lifted, a titanium plate was fixed in a decompressed position. A drainage tube was placed in every patient (Fig. 1).

**Fig. 2:**
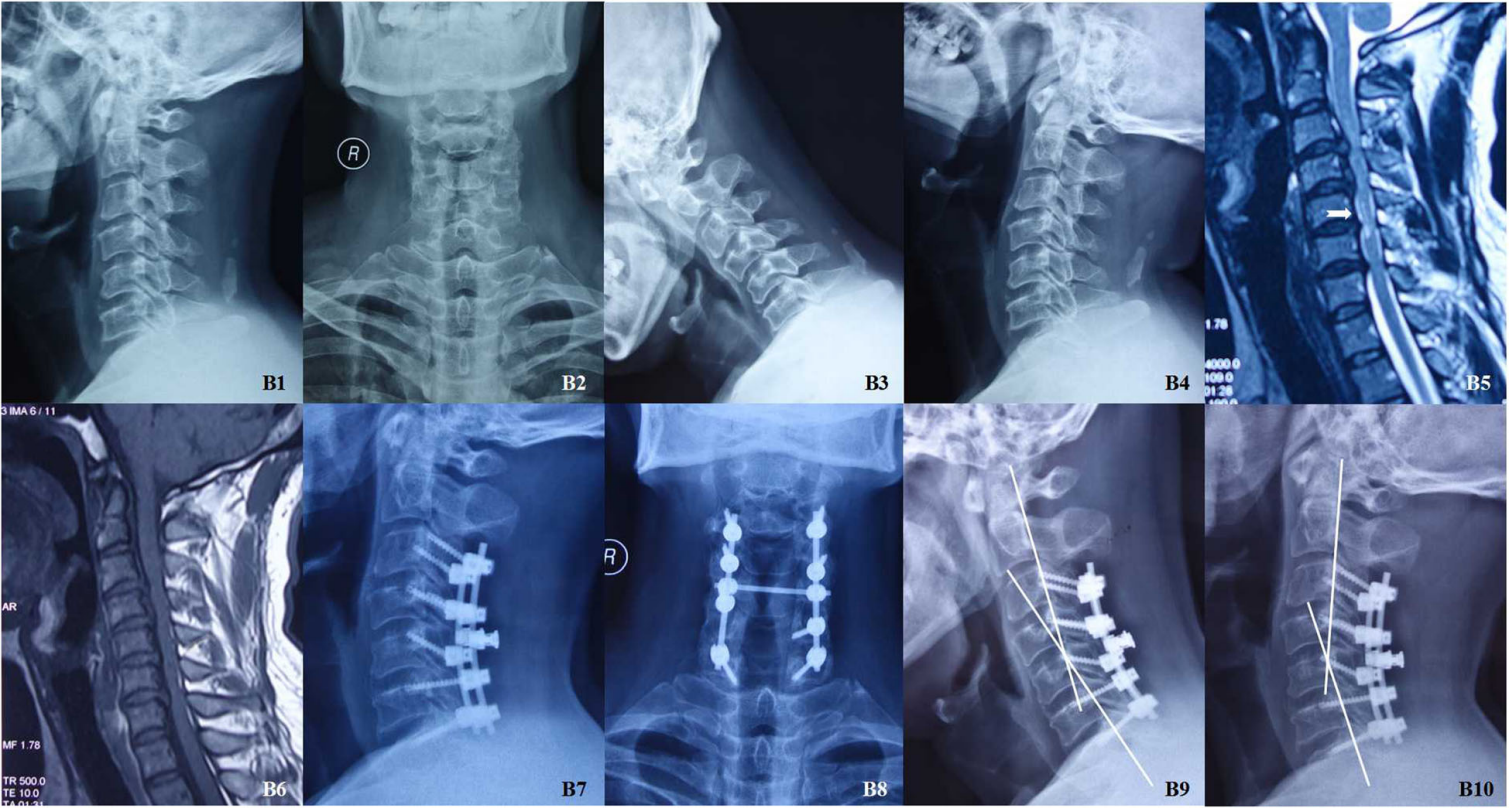
M, 47Y, multi-level cervical spondylotic myelopathy. Preoperative X-Ray images (B1 and B2); X-Ray images of extension and flexion (B3 and B4) shown no obvious cervical instability; Preoperative MRI scans (B5-B6) shown increased signal intensity on T2-weighted image (White arrow); Postoperative X-Ray images (B7 and B8); X-Ray images of extension and flexion at the 37th months after operation (B9 and B10) (White lines)

In the LF group, a standard midline posterior approach was used, and the decompression range was determined by the segments of stenosis. Laminectomy was performed after the holes for the lateral mass screws were drilled. The titanium screws were combined with curved titanium rods. The facet joints were decorticated, and autogenous bone was grafted along the lateral masses. Again, a drainage tube was placed in every patient (Fig. 2).

The C2 spinous process and the surrounding muscle were protected during the operation as far as possible [17]. All operations were performed by senior spine surgeons.

### Postoperative care

After the operation, the patients who underwent LF wore a brace for 6–8 weeks, while the patients who underwent LP wore a Philadelphia collar for 2 weeks. The drainage tube was removed when the drainage volume was less than 30 ml/24 h. By the third week after the operation, patients in both groups started neck muscle exercises.

### Statistical analysis

The statistical analyses were conducted using SPSS, version 22.0 (SPSS, Inc., Chicago, IL, USA). Statistical significance was achieved when *P* < 0.05, and all reported *P* values were two-tailed. The Kolmogorov–Smirnov test was used to evaluate the normality of the distribution of the obtained data, and the Levene test was used to test for homogeneity of variance. The Mann–Whitney U-test was used for processing non-normally distributed data. Paired and independent samples T-tests were performed to compare normally distributed data. The chi-squared test was performed to compare the operative level, smoking status, total complication rate, and sex.

## Results

There were 917 patients with MCSM with ISI on T2WI who underwent LF from January 2014 to June 2016. Among these, 99 patients were lost to follow-up, 18 patients also showed DSI on T1WI, 195 patients had cervical spine instability, 60 patients had a previous history of cervical surgery, 26 patients had other severe major organ dysfunction, and 116 patients had cervical kyphosis. Finally, there were 403 patients, of which 52 patients were paired by propensity score matching, forming the LF group.

Patients in the LF and LP groups were followed for 24–56 months (36.41 ± 11.19 months) and for 24–59 months (34.06 ± 8.31 months), respectively. There were no significant differences in the baseline features between the two groups (Table 1). The operative duration in the LF group was much higher than that in the LP group. At the final follow-up visit, there was no significant difference in the JOA score, VAS-N score, SF-36 MCS or SF-36 PCS between the two groups (Table 2). However, the extension and flexion ROMs of the cervical spine were much better in the LP group (Table 2). There were no cases of epidural hematoma or internal fixation failure in either group. There were two cases of superficial infection in the LF group, both of which healed well after the application of antibiotics. No complications, such as ‘re-closed door’ and kyphotic deformity, were found in the LP group at the final follow-up visit. In the LF group, seven screws in five patients were found to have unsatisfactory placement; however, there were no vascular and neurological injuries associated with misplaced screws. Six patients in the LF group and two patients in the LP group developed C5 nerve root palsy, but the symptoms completely disappeared after conservative treatment. Nine patients (5 in the LP group and 4 in the LF group) had AS, and there were 4 patients in the LF group with slight neck and shoulder discomfort at the final follow-up visit. One patient in the LF group had a dural tear. Two patients in LF group had adjacent segment degeneration (Table 2).

**Table 1:** The relevant data of patients and outcomes

Characteristics	LF Group	LP Group	*P* Value
Cases	52	52	
Age (years old)	59.62 ± 8.46	57.35 ± 7.18	0.14^a^
Sex (male/female)	24/28	27/25	0.56^b^
Tobacco use (yes/no)	20/32	23/29	0.55^b^
Operation levels	3.87 ± 0.67	3.79 ± 0.73	0.56^a^
HSIR	2.26 ± 0.31	2.38 ± 0.37	0.08^a^
Duration of pre-operative symptoms (months)	5.85 ± 2.72	5.21 ± 2.64	0.23^a^
With OPLL (yes/no)	19/33	14/38	0.29^b^
Operative Time (minutes)	158.17 ± 18.33	149.73 ± 15.56	0.01^a^
Intraoperative Blood Loss	225.84 ± 25.90	213.51 ± 47.98	0.11^a^
Pre-operation			
JOA	11.62 ± 1.21	12.03 ± 1.41	0.11^a^
VAS-N	4.04 ± 1.34	3.68 ± 0.92	0.11^a^
SF-36(PCS)	34.29 ± 13.87	37.05 ± 11.43	0.27^a^
SF-36(MCS)	36.76 ± 10.34	38.17 ± 11.59	0.51^a^
ROM of Extension and Flexion(°)	42.26 ± 12.35	45.88 ± 14.24	0.17^a^

*HSIR* high signal intensity ratio

*JOA* Japanese Orthopedic Association score

*OPLL* Ossification of the posterior longitudinal ligament

*VAS-N* Visual Analogue Score of Neck

*SF-36 (PCS and MCS)* The Physical and Mental Component Scores of the Short-Form 36

*ROM* Range of Motion

^a^T-test

^b^Chi-square test

**Table 2:** Complications and outcomes

Characteristics	LF Group	LP Group	*P* Value
Unsatisfactory Placement of Screw (case)	5	0	
C5 Nerve Root Palsyc (case)	5	2	
Axial Symptom (case)	3	5	
Superficial Infection (case)	2	0	
Dural tear (case)	1	0	
Adjacent segment degeneration	2	0	
Total Complications	18	7	0.012^
Final Follow-up			
JOA	13.90 ± 1.01	14.29 ± 1.12	0.07^#^
VAS-N	1.92 ± 0.94	1.73 ± 0.65	0.23^#^
SF-36(PCS)	47.24 ± 9.71	49.82 ± 9.04	0.16^#^
SF-36(MCS)	45.73 ± 7.76	48.99 ± 9.82	0.06^#^
ROM of Extension and Flexion(°)	5.23 ± 2.36	22.61 ± 6.75	< 0.001^#^

*JOA* Japanese Orthopedic Association score

*VAS-N* Visual Analogue Score of Neck

*SF-36 (PCS and MCS)* The Physical and Mental Component Scores of the Short-Form 36

*ROM* range of motion

^a^ T-test

^b^Chi-square test

## Discussion

MRI has been widely used in the clinical diagnosis of CSM, and it can clearly reveal the degree of spinal cord compression and intramedullary signal changes. Takahashi et al. [18] first described the phenomenon of ISI on MRI in patients with CSM and not only suggested that the presence of ISI can predict a poor prognosis but also noted that it may be related to spinal cord softening or secondary glial cell hypertrophy due to long-term spinal cord compression. Some studies have shown that preoperative ISI on T2WI may predict a poor prognosis after decompression surgery in patients with CSM [8, 9, 19, 20]. Ramanauskas et al. [21] divided the pathological changes of the spinal cord into three stages: early-stage changes, represented by spinal edema; middle-stage changes, represented by varying degrees of cystic necrosis of the central gray matter; and late-stage changes, represented by central cystic degeneration, syrinx formation, and atrophy. Early- and middle-stage patients were characterized by ISI on T2WI, and late-stage patients were characterized by ISI on T2WI and DSI on T1WI [21]. Ohshio et al. [22] found that abnormally high signal intensities appeared nonspecifically in mildly altered lesions or areas with edema on T2WI. LF and LP are the common treatments for MCSM [23]. A number of previous studies have proven that as a treatment option of MCSM, both methods can improve the symptoms and prevent further deterioration of the nervous system with no difference in efficacy [3, 11, 17, 23]. However, ISI on T2WI underwent laminoplasty has been reported to be associated with poor surgical outcomes [10]. For specific MCSM cases where pathological changes in the spinal cord have developed, it is still unclear whether preserving the mobility of the cervical spine will affect the recovery of neurological function in MCSM patients with or without ISI on T2WI.

In our study, 104 MCSM patients with ISI on T2WI who underwent LP or LF were retrospectively reviewed. To exclude interference, patients with DSI on T1WI were excluded, and there were no significant differences in the baseline features between the two groups (*P* > 0.05). In previous studies, symptom duration, smoking status, age, and the degree of neurological symptoms were considered risk factors for a poor surgical outcome [24, 25]. OPLL is a disease process characterized by progressive growth and calcification resulting in spinal canal compromise and serious neurological sequelae in advanced cases. A number of long-term studies have revealed both longitudinal and transverse disease progression in individuals treated both surgically and conservatively [26]. A long-term follow-up study reported by Iwasaki et al. [27] showed that only 1.5% of patients had neurological deterioration due to OPLL progression at the surgically treated levels and required additional surgery at the previously operated levels. They also found that the neurological recovery rate in patients with progression of the ossified lesion was not significantly different from that in patients without OPLL progression. Chiba et al. [28] also found that despite these high rates of radiographically documented progression, the rate of neurological decline in patients undergoing posterior surgery with LP was low.

At the final follow-up visit, patients in both groups achieved significant improvement in neurological function compared with preoperation. LP and LF both increased the volume of the spinal canal, relieved spinal cord compression, and did not cause cervical spine instability. Many studies have proven that LF and LP are both safe and effective [3, 4, 11, 17, 23]. Our results also show no significant difference in the JOA score, VAS-N score, SF-36 MCS or SF-36 PCS between the two groups at the final follow-up visit. In their respective systematic reviews, Bartels et al. and Phan et al. both found that the LF and LP approaches for MCSM result in similar clinical improvement [3, 11]. Additionally, a prospective multicenter study from the AOSpine North America and International Clinical Trial Research Network also showed that both LF and LP can significantly improve the neurological function and quality of life of patients with CSM, with no significant differences between the two methods [29]. These findings are consistent with the findings of the present research. According to our research results, preserving cervical spine mobility did not affect the recovery of neurological function. Henderson et al. and Yagi et al. found in their respective studies that tension and shear stress in the spinal cord increased during cervical flexion and extension, which may cause axonal injury and ISI on T2WI in the spinal cord [30, 31]. However, their results were obtained from patients with cervical instability. Rhee et al. found that fixation with titanium plates provided good cervical spine stability, allowing patients to have early postoperative activity [5].

The advantages of LF are considered to be adequate for decompression and restoring partial physiological curvature of the cervical spine. However, compared with LF, LP also could can achieve satisfactory decompression while preserving the complete posterior structure and cervical motion, improve the postoperative quality of life and the early rehabilitation of patients. A meta-analysis found out LP had fewer complications [11]. Lau et al. also reported LF was associated with greater blood loss and a higher long-term complication rate [2]. According to the results of this study at the final follow-up visit, the extension and flexion ROMs were decreased in both groups but were significantly better in the LP group than in the LF group, and the decompression levels still retained the ability to function during activity. Previous studies have confirmed that LP can retain the ROM of operated segments [3, 4, 23]. Our results show that as long as satisfactory decompression is achieved, preserving partial cervical spine motion does not affect neurological function recovery or quality of life.

Our results show that the operative duration was shorter in the LP group than in the LF group (*P* < 0.05). In the LF method, to increase the safety of the surgery, pedicle or lateral mass screws should be implanted cautiously, which together with intraoperative fluoroscopy, usually requires more time. On the other hand, LP is much easier and safer. In addition, proficiency in the surgical procedures also impacts the operative duration. Postoperative ‘re-closed door’ is a common complication of LP. Our results show no complications, such as ‘re-closed door’ and kyphotic deformity. This may be related to the use of micro-titanium plates. A long-term follow-up study of LP also demonstrated that the use of micro-titanium spacers could provide significant stability, which can prevent ‘re-closed door’ and preserve the physiological curvature of the cervical spine [32]. In our study, AS occurred postoperatively in some patients in both groups but were relieved in most patients approximately 3 months after the operation. The relieved AS were related to early-stage postoperative neck muscle training and the use of titanium plates. Edwards et al. also found that neck and shoulder muscle strength training could achieve pain relief [33]. Another study also confirmed that compared to suture suspension fixation, titanium plate fixation was able to decrease AS following LP [34]. As report, the average incidence rate of C5 nerve root palsy was 7.8% [35]. In our study, 7 patients exhibited C5 nerve root palsy, and the symptoms disappeared after conservative treatment within 3 months after the operation. C5 nerve root palsy is often associated with backward spinal cord drift. In our study, the incidence of C5 nerve root palsy in the LP group (2 patients) was lower than in the LF group (5 patients), but there was no significant difference. Michael et al. [29] also reported no significant difference in the incidence of C5 nerve root palsy between LF and LP. This difference could be attributed to the small sample size of our study. However, Li et al. [36] reported the lower incidence of C5 palsy and axial symptoms can be achieved by using LP compared with LF in their meta-analysis. In our study, the total complication rate was higher in the LF group than in the LP group (*P* < 0.05). Another meta-analysis also showed LP was associated with fewer complications [11].

Based on our experience and suggestions, LP was performed for patients without kyphosis and/or cervical spine instability and/or needing bilateral foraminotomy. LF was performed for patients with kyphosis and/or cervical spine instability and/or needing unilateral foraminotomy. And according to this research results, simple ISI on T2WI did not affect the recovery of neurological function. In consideration of shorter operative durations, lower complication rates and better extension and flexion ROMs, LP can be the first choice if the patient fit the indications for both procedures.

This study has some limitations. First, ISI on T2WI combined with DSI on T1WI has been widely reported is associated with poor surgical outcomes [31, 37]. Our study is limited by its sample size, and the patients with ISI on T2WI combined with DSI on T1WI were not included in the research. Future work will include this type of MCSM patient. Second, the K-line was used to predict the decompression effect in our study, and while postoperative MRI was not performed, the K-line is a useful predictive indicator for sufficient decompression by LP [13]. Finally, this was a short-term follow-up, retrospective study. The results require further validation and investigation in larger studies.

## Conclusion

The preliminary results show that as long as satisfactory decompression was achieved, preserving cervical vertebral mobility did not affect the recovery of neurological function. LP for MCSM with ISI on T2WI achieved similar clinical improvement as LF. However, longer operative durations, higher complication rates and lower extension and flexion ROMs were found in the LF group. These results require further validation and investigation in robust studies.

## Data Availability

The datasets generated and/or analysed during the current study are not publicly available due some patients refuse to disclose their personal disease details in publication, but all patients were agree to available from the corresponding author on reasonable request.
